# Microalbuminuria in patients with chronic kidney disease at Parirenyatwa Hospital in Zimbabwe

**DOI:** 10.11604/pamj.2013.14.39.1747

**Published:** 2013-01-28

**Authors:** Nyasha Chin'ombe, Ophius Msengezi, Hilda Matarira

**Affiliations:** 1 Department of Medical Microbiology, College of Health Sciences, University of Zimbabwe, Harare, Zimbabwe; 2 Department of Chemical Pathology, College of Health Sciences, University of Zimbabwe, Harare, Zimbabwe

**Keywords:** Microalbumin, microalbuminuria, chronic kidney disease, proteinuria, patients, Zimbabwe

## To the editors of the Pan African Medical Journal

Microalbuminuria (MAU), which is the presence of high levels of microalbumin in urine, has been linked to kidney damage or chronic kidney disease (CKD) [1, 2]. The presence of microalbumin in urine can therefore be an important marker for cardiovascular and renal risk in diabetes mellitus [3]. Although MAU has been known over the years to be an early predictor of CKD, its clinical relevance has not been explored widely in low-resource settings. The testing of MAU is also not available on a routine basis in their public health laboratories. In Zimbabwe, CKD is becoming a common occurrence. According to the Zimbabwe Kidney Foundation, the number of patients with CKD continues to go up every year. It is therefore important to detect the presence of this condition early so that corrective therapy can be instituted before it becomes chronic. In our study, we evaluated the presence of MAU in 70 patients with CKD attending Parirenyatwa Referral Hospital Renal Unit in Harare, Zimbabwe. They were selected based on the criterion that they had high plasma urea, creatinine, and electrolytes (sodium and potassium). The control subjects (n = 70) were recruited from the Zimbabwe National Army and Parirenyatwa Hospital staff and were apparently healthy with no known kidney problems. The demographic data were collected from both the patients and control subjects by administering a questionnaire with a set of questions. Patients and control subjects completed an informed consent form and the Medical Research Council of Zimbabwe approved the study protocol. The main equipments used in the study were the CX5 (Beckman, USA) and the COBA MIRA's Plus (Roche, USA). Reagents used included Bayer Multi-stick and microalbumin enzyme-linked immunosorbent assay (ELISA) kit (Randox, USA). The microalbumin kit was used according to manufacturer's recommendations. Microalbumin levels were measured from urine samples with anti-microalbumin antibody from the kit. Plasma urea, creatinine and electrolytes were evaluated at the Public Health Laboratory at Parirenyatwa Hospital (Zimbabwe) on the CX5 analyser. Proteins and nitrites in the urine were evaluated using the Bayer Reagent strips according to manufacturer's specifications. Results were analyzed using S plus software computer package.

Out of all the patients with CKD, there were 48 males (69%) and 22 females (31%). The age of the patients (all sexes) ranged from 20 to 74 years. The majority of them were in the 25-29 and 35-39 age-bands. The number of patients less than 30 years was 18 (13 males and 5 females). There were 24 males and 15 females who were between 30 and 50 years of age. The number of patients over 50 years of age was 13 (12 males and 1 female). The peak age-group (25-29 years) had 18 patients of which 8 were hypertensives (6 males and 2 female college students). The 35-29 peak had 17 patients and 13 of these had hypertension. The incidence of renal diseases was higher in males compared to females. The aetiological clinical conditions of the CKD patients were also evaluated. They included hypertension, type 2 diabetes mellitus, glomerulonephritis, HIV infection with hypertension, kidney transplanted, hypertension with diabetes, nephrotic syndrome and cancers. The majority of the patients (58%) had hypertension. There were also other conditions such as small kidney, tuberculosis infection, urinary tract obstruction, history of schistosomiasis infection, systemic lupus erythematosus (SLE) with hypertension, malaria infection, malignancy with hypertension and traditional African herbal poisoning which were found in the patients.

The mean levels of microalbumin in CKD patients and healthy control subjects were 157 ± 71 mg/l and 6.6 ± 5.0 mg/l respectively and there was a significant difference between the two groups (p < 0.001). Out of all the 70 renal patients 44 (63%) had microalbumin as high as 210 mg/l. Of these 44 patients, 29 were hypertensive, 3 had diabetes with hypertension, 2 had diabetes alone, 2 had glomerulonephritis, 1 had hypertension and hepatitis, 1 had previous traditional African herbal poisoning, 1 had obstruction of the urinary tract, 1 had secondary to malaria, 1 was HIV-positive and had hypertension, 1 had nephrotic syndrome, 1 had tuberculosis and 1 had SLE with hypertension. Of all the patients, only 5 had microalbumin within the reference range (0-26 mg/l) and 21 patients had microalbumin within the 26-199 mg/l range. All the controls subjects (n = 70) had microalbumin within the normal reference range. Other biochemical tests to evaluate the levels of creatinine, sodium, potassium, urea, nitrite and hemoglobin in patients were performed. The levels of serum creatinine in CKD patients were above those of the control subjects. Four patients had less than 300 mmol/l, 10 had 300-600 mmol/l, 15 had 600-900 mmol/l, 20 had 900-1200 mmol/l, 16 had 1200-1500 mmol/l and 5 had 1500-1800 mmol/l levels of creatinine. The highest frequency was at 1 200 mmol/l peak. The mean sodium level in the renal group was 139 ± 5 mmol/l and was significantly above the mean levels in control group (p < 0.001). The mean potassium level in CKD patients was also significantly above that of the control group (p < 0.001). The same trend was found with urea and hemoglobin levels. The evaluation of urine nitrites and proteins was qualitative. Urine nitrites were negative in 87% of the patients. They were 61 nitrite-positive and 9 -negative patients in this study. Urine proteinuria was positive in 67% of the patients.

Since CKD normally progresses to end-stage kidney disease which requires occasional haemodialysis or kidney replacement therapy by transplantation, early detection is therefore important in order to circumvent total renal failure and mortality. The presence of microalbumin in urine is a now recognized as a risk marker for CKD and its complications in humans [1, 4–9]. The commonest clinical condition in CKD at Parirenyatwa Referral Hospital was hypertension. Hypertension was present in more than half of the patients. Previous studies have shown that the prevalence of hypertension in Zimbabwe was very high, especially in urban dwellers [10, 11]. This could possibly explain why there were many patients with kidney failure in our study with hypertension. In our study, the number of hypertensives was followed by those with diabetes. Previous studies in Zimbabwe have also shown a link between insulin resistance and hypertension [12, 13]. All these people with hypertension and diabetes are at high risk of developing CKD. High blood pressure (hypertension) and diabetes were very prevalent in patients in our study and could be the primary causes of CKD in Zimbabwe. Other minor interesting clinical conditions found in the renal patients included HIV infection with hypertension, small kidney, tuberculosis infection, urinary tract obstruction, history of schistosomiasis infection, malaria infection, malignancy with hypertension and traditional African herbal poisoning. There are few studies to link these conditions with kidney disease. Most people in Zimbabwe use African herbal medicines and several cases of poisoning have been reported [14, 15]. These herbal concoctions are poorly formulated and can cause kidney damage especially due to over-dose. Zimbabwe has a high incidence of Plasmodium falciparum malaria and there is an association between malarial parasitic infection and CKD [16, 17]. Infection by Schistosoma mansoni is also known to cause kidney disease and this parasite is prevalent in Zimbabwe [18, 19]. Schistosomiasis is common in most parts of Zimbabwe [18, 19]. In this study, there was a very small proportion of the patients (4%) who were HIV-positive. HIV-1 and TB infections are high in Zimbabwe, but their links with kidney disease have not previously been investigated. However, HIV infection is sometimes associated with a wide spectrum of kidney complications such as acute kidney injury, electrolyte and acid-base disturbances, HIV-associated glomerular disease and adverse side effects related to anti-retroviral treatment [20]. In this study, we did not investigate the genetic link between the CKD and ethnicity. All the patients recruited in the study were indigenous black Africans. The high prevalence of kidney disease in black indigenous Africans has previously been linked to genetic variants in the MYH9 gene that encodes non-muscle myosin 2a protein [21]. Defects in this gene have also been associated with greater risk of kidney disease in African-American population [22, 23].

The indices of altered renal function (microalbumin in the urine and increased serum creatinine concentration) were evaluated in the CKD patients in this study. Unlike the normal subjects, the majority of the patients had MAU. More than half of the patients had overt MAU. This illustrated an association of CKD and MAU. Only one study was previously done in Zimbabwe to determine the prevalence of microalbuminuria in hypertension patients [11]. The levels of microalbumin in urine were found to be significantly higher in patients with hypertension than normal subjects [11]. Other studies have also found an association of MAU and CKD [3, 4, 24]. The association of MAU and CKD with other metabolic conditions such as hypertension and diabetes mellitus has also been reported [5, 6, 8]. This is in agreement with the findings of our study.The levels of the routine chemistries (creatinine, hemoglobulin, sodium, potassium and urea) in our study were consistent with the expected pattern in CKD and were significantly different from those of the control group. Creatinine, which is a good marker or indicator of kidney function was very high. The distribution of haemoglobin showed the presence of anaemia in most patients with CKD. This is in agreement with what other investigators found [23]. The wide differences between renal patients and control ranges showed that haemoglobin could also be a good predictive marker for CKD. The majority of patients had proteinuria. Proteinuria, just like MAU, is also a good marker of kidney dysfunction [24]. In conclusion, MAU should be used as marker of marker of renal dysfunction in CKD in low-resource settings such as Zimbabwe. It may also be used as a marker for other metabolic problems associated with CKD.
